# Analysis of *Caenorhabditis elegans* Sperm Number, Size, Activation, and Mitochondrial Content

**DOI:** 10.21769/BioProtoc.4035

**Published:** 2021-06-05

**Authors:** Amy M. Hammerquist, Chia-An Yen, Sean P. Curran

**Affiliations:** 1 Leonard Davis School of Gerontology, University of Southern California, Los Angeles, United States; 2 Department of Molecular and Computational Biology, Dornsife College of Letters, Arts and Sciences, University of Southern California, Los Angeles, United States; 3 Norris Comprehensive Cancer Center, Keck School of Medicine, University of Southern California, Los Angeles, United States

**Keywords:** C. elegans, Fertility, Sperm quality, Spermatogenesis, Mitochondria

## Abstract

Infertility is a widespread and often unexplained issue. Studying reproduction using *C. elegans* males offers insight into the influence of individual factors on male fertility in humans. We have created a collection of protocols to assess several aspects of *C. elegans* sperm quality, including number, size, rate of activation, and mitochondrial morphology. Studying sperm biology in a model system such as *C. elegans* allows access to the wealth of resources and techniques that have been optimized for that organism while providing valuable biological information that may be applicable to other systems.

Graphic abstract:

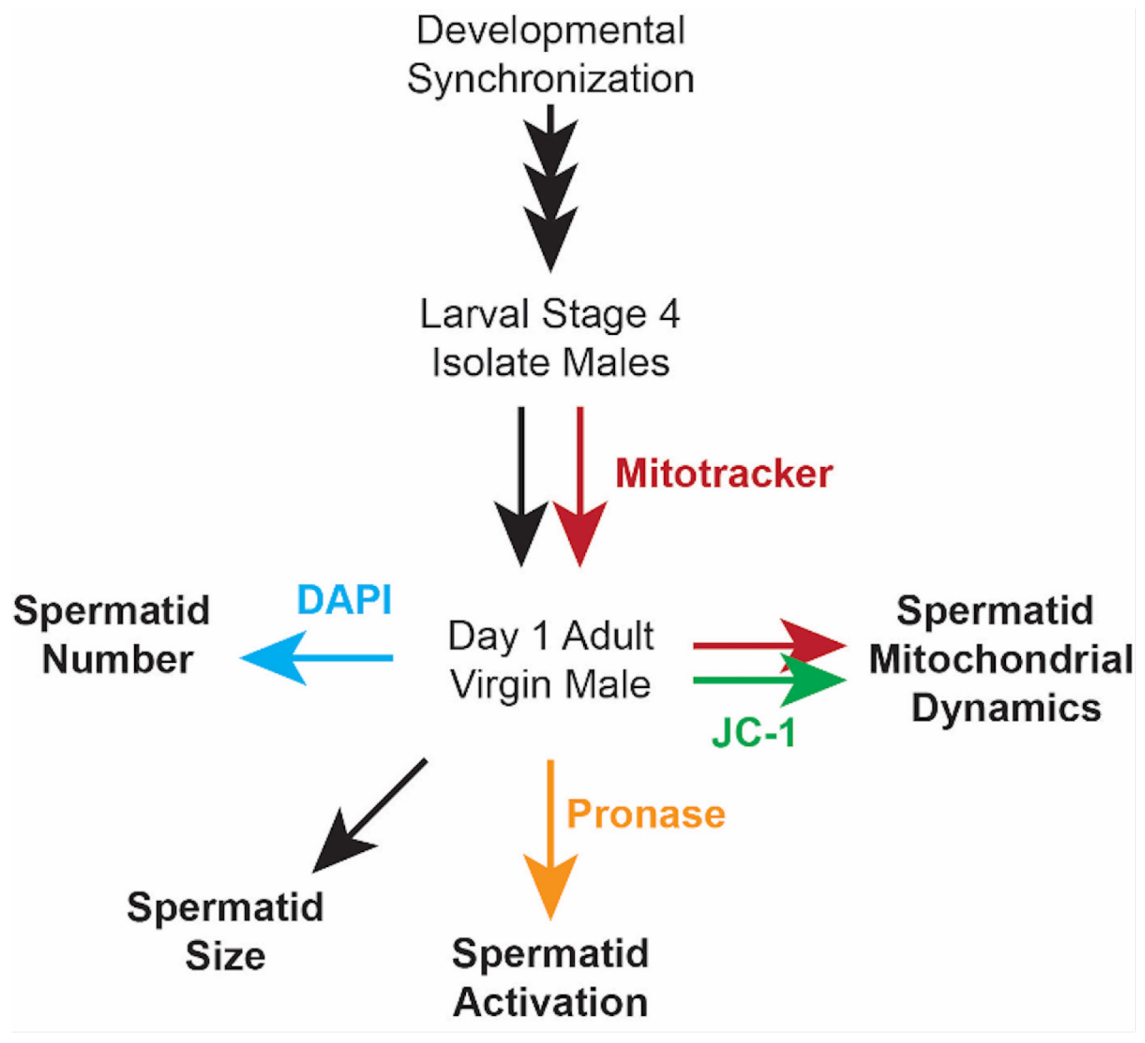

Flowchart depicting the preparation of *C. elegans* males and subsequent sperm quality assays

## Background

Approximately 8% of couples worldwide suffer from infertility (World Health Organization, 1987), and 30% of those cases have unknown causes (Practice Committee of the American Society for Reproductive, 2006). The wide variety of factors that affect male fertility, both genetic and environmental (Oliva *et al.*, 2001; Huynh *et al.*, 2002), make studies in humans susceptible to confounding influences. Model systems can be useful in investigating the influence of single factors on various aspects of fertility. *Caenorhabditis elegans* is a genetically tractable organism with a defined number of cells and germ cells that can be examined phenotypically.

Studies of mammalian mitochondrial function (including mitochondrial morphology, mitochondrial genome and copy number, mitochondrial protein levels, and enzyme activity of the electron transport chain complexes) have demonstrated a link between mitochondrial health and sperm function (Amaral *et al.*, 2013). Additionally, several mitochondrial mutants negatively impact *C. elegans* fertility, although the exact mechanism of action of these pathways is unknown (Jonassen *et al.*, 2002; Grad and Lemire, 2004). Recent work from our lab has demonstrated that *mafr-1*, a regulator of RNA polymerase III (Hammerquist and Curran, 2020), and *alh-6* and *prdh-1*, enzymes in the mitochondrial proline catabolism pathway (Yen *et al.*, 2020; Yen and Curran, 2021), are required for proper sperm function in *C. elegans*, and these roles may be conserved in mammalian models (Bonhoure *et al.*, 2015; Yen and Curran, 2021).

We recently developed and published methods to assess various metrics of spermatid quality in *C. elegans* (Hammerquist and Curran, 2020; Yen and Curran, 2020; Yen *et al.*, 2020). Some of the methods we present, albeit with added details and refined techniques, are largely unchanged from their original publication decades ago (Ward *et al.*, 1983; Shakes and Ward, 1989; LaMunyon and Ward, 1998), whereas others, specifically the quantification of spermatid mitochondrial fusion, were first developed in our lab (Yen *et al.*, 2020). Here, we provide detailed protocols for a comprehensive set of assays that measure the quality and mitochondrial health of spermatid.

## Materials and Reagents

1. Microscope slides (VWR, catalog number: 48312-004)
2. Sterile 6 cm Petri dishes (VWR, catalog number: 25373-085)
3. Platinum wire (Tritech Research, catalog number: PT-9010)
4. Eyelash brush (consisting of a human eyelash attached to a Pasteur pipette, generic, with tape, generic)
5. Microscope slide cover slips (VWR, catalog number: 48366-227)
6. PAP pen (Liquid Blocker, catalog number: Z377821)
7. Plastic or glass container that can be sealed (Glasslock, catalog number: OCRT-048)
8. Paper towels (generic)
9. Parafilm (Sigma-Aldrich, catalog number: P7543)
10. 25G needles (BD, catalog number: 305125) or scalpel (Technocut, catalog number: 6008T-10)
11. 750 ml centrifuge bottles that can be sterilized by autoclaving (generic)
12. 2-200 μl micropipette tips (Genesee Scientific, catalog number: 24-151RL)
13. *E. coli* OP50-1 (available from *Caenorhabditis* Genetics Center)
14. Worm strains (available from *Caenorhabditis* Genetics Center)
15. Sodium Chloride (Fisher Scientific, catalog number: 02-004-047)
16. Peptone (BD, catalog number: 211820)
17. Bacto Agar (BD, catalog number: 214040)
18. Cholesterol (Sigma-Aldrich, catalog number: C8667)
19. Ethanol (VWR, catalog number: 89125-172)
20. Calcium Chloride (Sigma-Aldrich, catalog number: C3881)
21. Magnesium Sulfate (Sigma-Aldrich, catalog number: M2773)
22. Potassium Phosphate dibasic (Sigma-Aldrich, catalog number: P5504)
23. Potassium Phosphate monobasic (Sigma-Aldrich, catalog number: P0662)
24. Streptomycin sulfate (Sigma-Aldrich, catalog number: S6501)
25. LB powder (Teknova, catalog number: L9315)
26. HEPES (J.T. Baker, catalog number: 4018-04)
27. Potassium Chloride (Sigma-Aldrich, catalog number: P3911)
28. BSA (Sigma-Aldrich, catalog number: A9647)
29. Dextrose (J.T. Baker, catalog number: JT1919)
30. Sodium Phosphate dibasic (Sigma-Aldrich, catalog number: S9763)
31. Potassium Chloride (Sigma-Aldrich, catalog number: P3911)
32. Triton X-100 (Sigma-Aldrich, catalog number: X100-100 ml)
33. Isopropanol (BDH, catalog number: BDH1133)
34. DAPI (Sigma-Aldrich, catalog number: D9542)
35. Petroleum jelly (Vaseline)
36. Nail polish (clear, generic)
37. Immersion oil for microscope (Fisher Scientific, catalog number: 12-624-66B)
38. Vectashield mounting medium (VWR, catalog number: H-1000)
39. Pronase (Millipore Sigma, catalog number: 53702)
40. MitoTracker^TM^ Red CMXRos (Thermo Fisher, catalog number: M7512)
41. MitoProbe^ TM^ JC-1 Assay Kit (Thermo Fisher, catalog number: M34152)
42. Nematode Growth Medium (NGM) plates (see Recipes)
43. Phosphate buffered saline (PBS) (see Recipes)
44. PBS + 0.01% Triton X-100 (PBST) (see Recipes)
45. SM Buffer (dextrose) (see Recipes)
46. SM Buffer (BSA) (see Recipes)
47. M9 (see Recipes)
48. DAPI stock solution (see Recipes)
49. Pronase working solution (see Recipes)
50. MitoTracker stock solution (see Recipes)
51. JC-1 stock solution (see Recipes)

## Equipment

1. 2-20 μl micropipette (Gilson FA10003M)
2. 20-200 μl micropipette (Gilson FA10005M)
3. Scale (generic)
4. pH meter (generic)
5. Microcentrifuge for 1.5 ml tubes (Eppendorf, model: 5430)
6. Refrigerated centrifuge with swing bucket rotor for 750 ml centrifuge bottles (Beckman Coulter, model: Allegra X-15R)
7. Tube rotator (Thermo Scientific, catalog number: 88881001)
8. Bunsen burner (generic)
9. Dissecting microscope (Nikon SMZ 800 with fiber optic diascopic illumination stand)
10. Compound microscope with DIC, DAPI, GFP, and RFP filters, and 10×, 40-63×, and 100× objectives (Zeiss, model: AxioScope5)
11. Color camera (Zeiss AxioCam MRm)
12. Worm incubator (Generic, maintain at 20 °C)

## Software

1. Imaging software
  Dependent on the microscope used (in this case, we used a Zeiss AxioScope and the associated ZEN imaging software)
2. ImageJ (available from NIH)
3. Data analysis software
We use GraphPad prism, although any software capable of *t*-test will suffice. ANOVA may be helpful if comparing multiple conditions but not strictly necessary.

## Procedure

- Prepare slides (to be used in D. Sperm size assay; E. Sperm activation assay; F. Qualitative mitochondrial morphology assay; and G. Quantitative mitochondrial fusion assay).
  1. Using a PAP pen, draw circles of 1-1.5 cm in diameter on the microscope slides.
*Note: One standard slide will easily fit two grease circles, with room to cover each individually (when using 22 × 22 mm square cover slips).*
  2. Allow to dry overnight (or > 2 h in lamellar flow hood).
- Prepare males
  1. Maintain the growth of males in the strains needed for the assay.
*Notes:*
    - *Thirty to forty males are needed for sperm number assay; at least five males are needed per replicate of sperm size, activation, and mitochondrial assays. This number is easily obtained from a mixed population of males and hermaphrodites. To ensure the propagation of males in the strains used, transfer young adult males and hermaphrodites (3:1 ratio) to fresh plates 4-5 days prior to picking males for the assay.*
    - *If mating ability and/or male fertility is impaired, males should be mated to hermaphrodites (3:1 ratio) overnight on NGM plates seeded with 20 μl of 25× OP50-1 (see Recipe 1i Note for further detail) and transferred to stock NGM plates. The smaller food spots increase the frequency of encounters between males and hermaphrodites, thus increasing the likelihood of successful mating. Mate animals 4-5 days prior to picking males for the assay.*
    - *
If larger numbers of animals are desired, populations can be synchronized by alkaline hypochlorite treatment of a mixed population of males and hermaphrodites as described before (Yen and Curran, 2020). To ensure you have enough males, only use populations with approximately 50% of males.
*
  2. Pick larval stage 4 (L4) virgin males to stock NGM plates free of hermaphrodites on the day prior to assay (consult WormAtlas (Altun *et al.*, 2021) for further information on identifying L4 males).
*Notes:*
    - *Picking L4 males ensures that premature activation does not occur via mating prior to the assays. Males should be prepared in this way for all assays, except for qualitative mitochondrial morphology, for which the MitoTracker dye should be applied to food prior to transferring males to the plates (see Procedure F).*
    - *Males tend to crawl off plates in search of hermaphrodites if isolated for too long; if this becomes a problem, some loss of males can be prevented by allowing hermaphrodites (preferably gravid, unmated ones) to crawl on seeded NGM to scent it with pheromones and removing them before transferring males to plate.*
  3. Incubate for 18-24 h at 20 °C.
- Sperm number assay
  1. Wash male worms from isolation plate with PBS + 0.01% Triton X-100 (PBST) in a 1.5 ml tube.
  2. Centrifuge for 1 min at 560 *× g.* Remove supernatant to 0.1 ml (use marks on the tube as a guide).
  3. Wash 1-2 additional times with PBST.
*Note: These washes remove E. coli from the worms to be stained. Wash until the supernatant removed is clear (and not cloudy with bacteria). Do not wash beyond this point. Additional washes increase the likelihood of losing worms while aspirating the supernatant.*
  4. Centrifuge for 1 min at 560 *× g.* Remove supernatant to 0.1 ml.
  5. Add 600 μl of 40% isopropanol and rotate for 3 min at room temperature to fix worms.
  6. While worms are fixing, make staining solution: dilute 1 mg/ml DAPI stock solution to 10 μg/ml in 40% isopropanol (10 μl DAPI per 1 ml 40% isopropanol).
  7. Centrifuge for 1 min at 560 *× g.* Remove supernatant to 0.1 ml.
  8. Add 600 μl of staining solution and incubate in the dark for 5 min.
  9. Centrifuge for 1 min at 560 *× g.* Remove supernatant to 0.1 ml.
  10. Add 600 μl PBST and incubate in the dark for 30 min to destain.
  11. Mount samples on microscope slides (regular slides, not those prepared in Procedure A) with Vectashield mounting medium.
  12. Cover with a cover slip and seal the edges with nail polish. Allow to dry (in the dark) for 5-10 min.
  13. Image at 40-63× with DIC and DAPI filters, ensuring all spermatids are visible in each worm.
*Note: Spermatids should be restricted to the seminal vesicle in male worms and can be distinguished from other germ cells by their compact nuclei.*
  14. Collect z-stacks of each seminal vesicle, ensuring all spermatids are captured (use 40*×* if needed to zoom out to include all spermatids). A representative image is shown in Figure 1.
*Note: Set bounds at upper and lower edges of the seminal vesicle (where the first spermatid is visible on each side of the seminal vesicle) and image at a z-plane distance of 0.25 microns. This distance is determined by the auto settings of the Zeiss imaging software.*
    **Figure 1.**
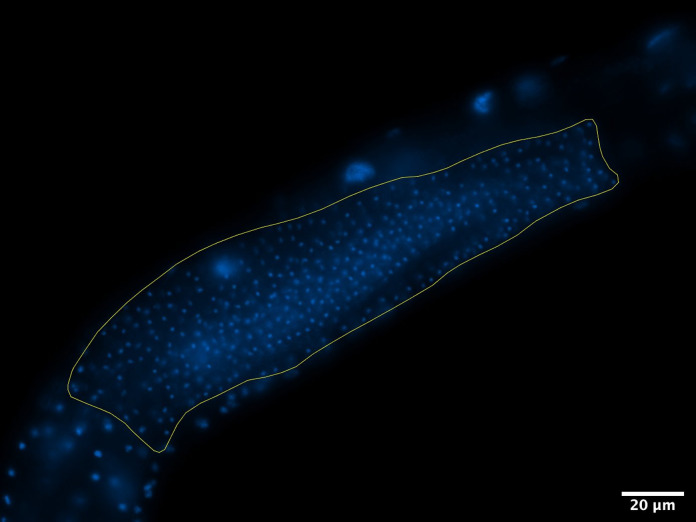
DAPI-stained spermatids in the seminal vesicle. The worm is positioned with the anterior region to the left and posterior to the right. The seminal vesicle is outlined in yellow. Scale bar, 20 μm.
- Sperm size assay
  1. Clean worms to get rid of *E. coli.*
    Cleaning can be done in one of two ways. For the first method, pipet two drops of ~15 μl M9 onto a Petri dish lid and use a platinum wire worm pick to transfer 5 day one adult virgin males to a drop of M9. Using an eyelash brush, transfer the worms into the second M9 drop before moving them into the SM buffer on the prepared slide. For the second method, use a platinum pick to transfer >5 day one adult virgin males to an unseeded NGM plate and allow them to crawl from the food spot. Then use a clean platinum pick (without bacteria) to transfer 5 males from the unseeded plate to the SM buffer on the prepared slide. Carefully move the worms individually at each transfer step. Picking up worms without food requires practice and is done in a scooping motion where the pick goes below the worm.
  2. Pipet 35 μl of SM buffer containing dextrose into the grease circle on the prepared slide (from Procedure A).
  3. Transfer 5 clean virgin males to the SM buffer on the slide using either an eyelash brush or a platinum pick, depending on how the animals were cleaned (see Step D1).
  4. Under a dissecting microscope, dissect worms to release the sperm.
    - Using a scalpel or two 25-gauge needles (slide flat surfaces of needles across each other mimicking scissor blades), slice males between the midpoint and tail (approximately 1/3 body length from the tail).
*Note: Move rapidly as dissected sperm may be sensitive to changes in salt concentration and/or pH resulting from evaporation.*
    - Sterilize the scalpel or needles between samples by dipping in ≥ 70% ethanol and passing through a Bunsen burner flame. Allow to cool before use.
  5. Cover dissected spermatids with a cover slip.
  6. Seal with nail polish.
  7. Image with a 100*×* objective using a DIC filter. A representative image is shown in Figure 2.
    - Complete imaging as quickly as possible because spontaneous activation has been observed when isolated spermatids are left for prolonged periods. In experienced hands, this should take no more than 5 min.
    - Score only spherical cells and at least 100 per replicate.
*Note: Typically, 100 spermatids are easy to obtain from one slide of five dissected day one adult males. This is attainable from imaging approximately 5-10 fields. The number of fields imaged depends on how the dissection spreads them on the slides. It may help to keep a rough mental count on the number of spermatids while imaging; always take a few extra images.*
    **Figure 2.**
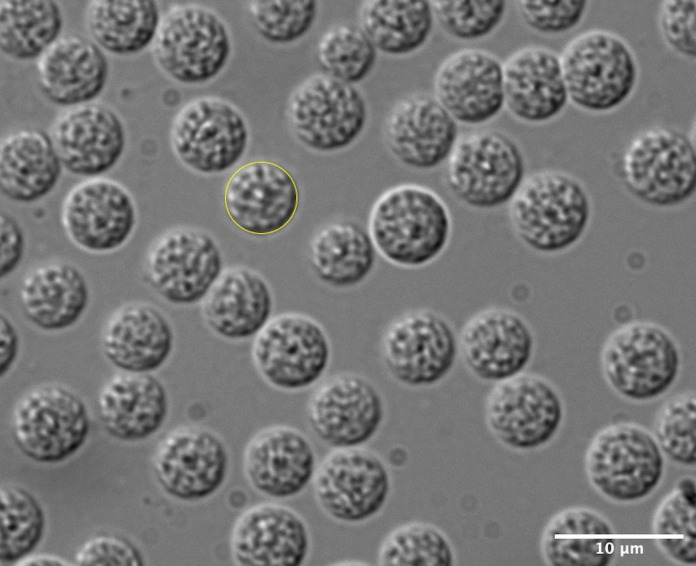
DIC image of isolated spermatids for size analysis. One spermatid has been outlined (in yellow) in ImageJ for size analysis. Scale bar, 10 μm.
- Sperm activation assay
  1. Prepare a humid chamber (Figure 3)
    - Place a wet paper towel on the bottom of a lidded container. Cover with a sheet of parafilm to create a dry surface to place slides on.
    - Cover with the lid.
    **Figure 3.**
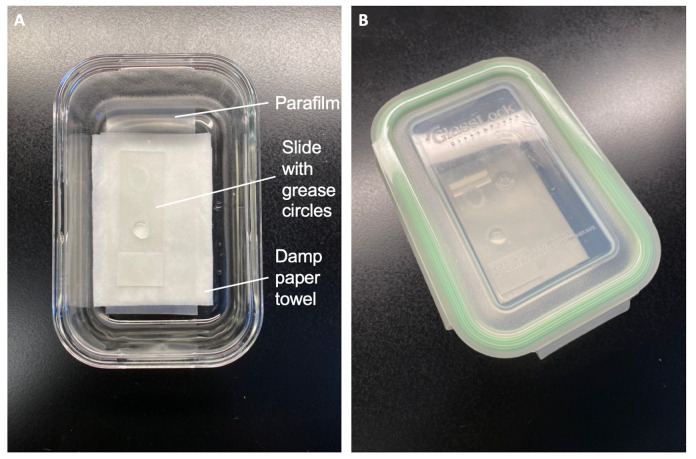
DIY humid chamber in open (A) and closed (B) configurations
  2. Prepare the Pronase working solution (see Recipes) and SM containing BSA and 400 ng/μl of Pronase.
    After the Pronase working solution is completely dissolved, dilute 4 μl in 96 μl of SM buffer containing BSA.
  3. Clean worms to remove *E. coli* (see Procedure D for details).
  4. Pipet 35 μl of SM buffer containing BSA into the grease circle on the prepared slide (from Procedure A).
*Note: Activation assays are done using SM buffer containing BSA rather than Dextrose. We have tested with both supplements and noted that BSA is needed for activation.*
  5. Transfer 5 clean virgin males to the SM buffer on the slide using either an eyelash brush or a platinum pick, depending on how animals were cleaned (see Step D1a).
  6. Under a dissecting microscope, dissect worms to release the sperm (see Procedure D for instructions).
  7. After dissecting the final male, add 35 μl of SM buffer containing BSA and 400 ng/μl Pronase.
  8. Incubate in the humid chamber (covered) for 15 min.
*Note: In our hands, 15 min activates approximately 80% of WT spermatids, but the activation is sensitive to multiple factors, including handling time, temperature, and freshness of buffer components. The incubation length may need to be determined empirically on an individual basis.*
  9. During the incubation, prepare the cover slip.
    - Using forceps or a pipette tip, create a thin line of petroleum jelly around the edges of the coverslip.
*Note: We have found that sealing the slides with nail polish affects activation and thus use only petroleum jelly to seal slides for this assay.*
  10. After 15 min, cover the dissected animals with the prepared cover slip. Do not seal further.
  11. Image with a 100× objective using a DIC filter. A representative image is shown in Figure 4.
*Notes:*
    - *Because sperm may be in different focal planes, and pseudopods of activated spermatids may be difficult to see in a single image, we recommend imaging each field of view in two different focal planes to aid in scoring (this distance need not be precisely measured). The second image serves as an aid in determining the activation status of spermatids that are unclear in the first one. Often, the movement of pseudopods in the time between capturing the images also helps reveal details that might not be evident in a single image.*
    - *Because the cover slip is adhered to the slide using only surface tension, changing objectives after oil immersion may cause it to shift or come off completely. For this reason, examine the slide using a lower magnification (non-oil immersion) objective before imaging. Do not return to a lower power objective after immersion oil is applied.*
    - Ensure all imaging is complete within the first 5 min after the incubation is complete (20 min after adding SM/Pronase solution).
    - Score at least 100 spermatids per replicate.
    **Figure 4.**
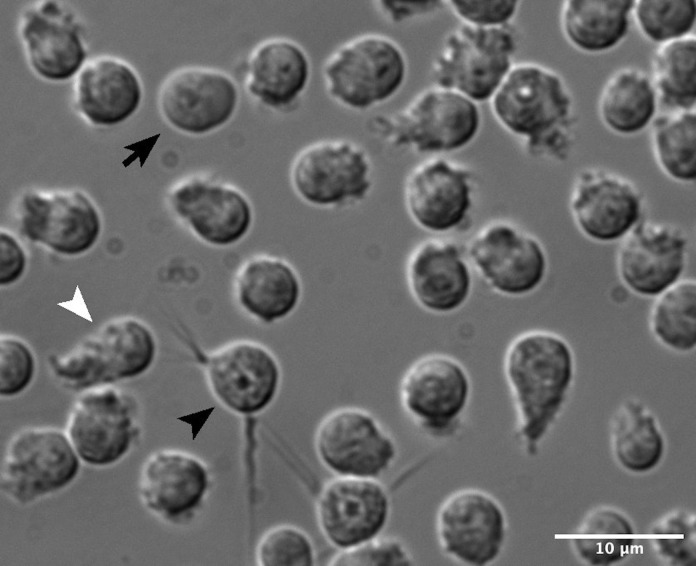
DIC image of sperm isolated for activation assays at various states of activation, showing spike (black arrowhead), lobed (black arrow), and full pseudopod (white arrowhead) projections. Please refer to Figure 2 for inactive (round) spermatids. Scale bar, 10 μm.
- Qualitative mitochondrial morphology assay
  1. Dilute the MitoTracker stock solution (1 mM in DMSO) to 100 μM in M9.
  2. Add 50 μl of working MitoTracker solution to the food spot of NGM plate seeded with 50 μl of 25× concentrated OP50 and let dry.
  3. Transfer L4 males to the prepared plate (from the previous step) using either an eyelash brush or a platinum pick, depending on how animals were cleaned (see Step D1).
  4. Incubate for 18-24 h at 20 °C.
  5. Clean worms to remove *E. coli* (see Procedure D for details).
  6. Pipet 35 μl of SM buffer containing dextrose into the grease circle on the prepared slide (from Procedure A).
  7. Under a dissecting microscope, dissect worms to release the sperm (see Procedure D for instructions).
  8. Cover the dissected spermatids with a cover slip.
  9. Seal with nail polish.
  10. Image with a 100*×* objective using DIC and AlexaFluor594 filters (RFP channels should also work). Representative images are shown in Figure 5.
    - Ensure imaging is complete within 10 min.
    - Score only spherical cells.
    - Score at least 100 spermatids per replicate.
    **Figure 5.**
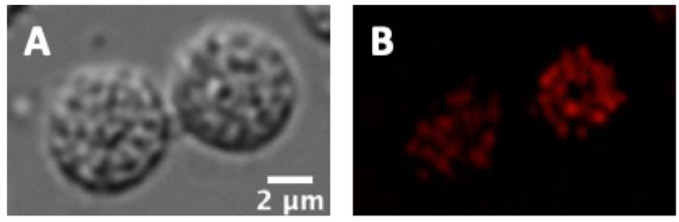
MitoTracker-stained spermatids in DIC (A) and AF594 (B) channels. Scale bar, 2 μm.
- Quantitative mitochondrial fusion assay
  1. Prepare a humid chamber (see Procedure E for details).
  2. Clean worms to remove *E. coli* (see Procedure D for details).
  3. Prepare staining solution.
    - Add 8 μl of 1.5 mM JC-1 stock to 792 μl of SM containing BSA (15 μM final concentration).
  4. Pipet 35 μl of SM buffer containing BSA with JC-1 into the grease circle on the prepared slide (from Procedure A).
  5. Transfer 5 cleaned virgin males to the SM buffer on the slide using either an eyelash brush or a platinum pick, depending on how animals were cleaned (see Step D1a).
  6. Under a dissecting microscope, dissect worms to release the sperm (see Procedure D for instructions).
  7. After dissecting the final male, add 25 μl of SM buffer containing BSA and JC-1.
  8. Incubate in the humid chamber (covered) for 10 min.
  9. Wash three times with 100 μl of SM buffer containing BSA (no JC-1).
    - Be careful to avoid completely removing the liquid from dissected spermatids, as this will affect spermatid physiology (and therefore mitochondrial phenotypes).
    - Be careful to avoid overflowing the grease circle and losing the sample. If necessary, use smaller wash volumes (80-90 μl).
  10. Cover the dissected animals with a cover slip and seal with nail polish.
  11. Image with a 100*×* objective using AlexaFluor488 (Green) and AlexaFluor546 (Red) channels. Representative images are shown in Figure 6.
*
Note: JC-1 is a mitochondria-specific dye that reflects changes in membrane potential. The ratio of red fluorescence (high membrane potential mitochondria species) to green fluorescence (low membrane potential mitochondria species) is a commonly used metric of mitochondrial health (Sivandzade et al., 2019).
*
    - Collect z-stacks of spermatids to be scored.
*Note: Collect 8-9 images with a spacing of 0.8 microns in the z-plane.*
    - Ensure imaging is complete within 10 min, as spermatids are alive (not fixed) and can undergo mitochondrial changes.
    - Score only spherical cells.
    - Score at least 40 spermatids per replicate.
  **Figure 6.**
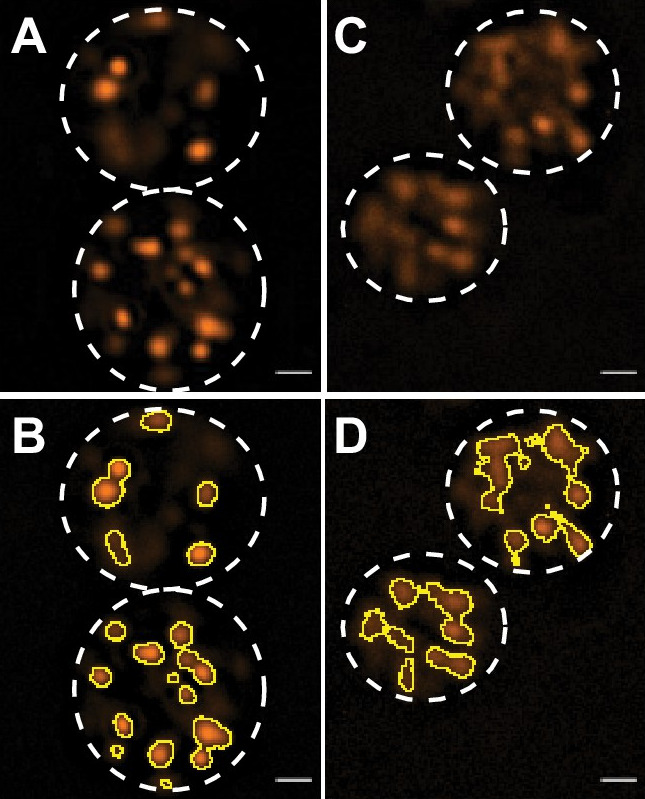
JC-1-stained spermatids from wild type (A-B) and *alh-6* mutant (C-D) animals, showing mostly spherical and oblong mitochondria, respectively. (B and D) ImageJ detection of JC-1 stained sperm mitochondria. Spermatid Outlines are marked with white dashed lines. Scale bar, 1 μm. This image was previously published in Yen *et al.* (2020).

## Data analysis

- Sperm number assay
  1. Deconvolve z-stacks.
  2. Count spermatid nuclei in z-stacks.
*Note: To avoid counting the same nuclei more than once or losing track of the ones counted, divide the seminal vesicle into multiple sections by drawing lines and counting one section at a time. For reference, in a 40× stack of images for one seminal vesicle, we selected 50-80 sections. The number of sections will depend on how the worm lays and how much mounting medium is between the cover slip and the slide (the smaller the volume, the flatter the worms may be, and fewer z-stacks will be needed to acquire the image, whereas more counting sections may be needed).*
  3. Each replicate should consist of at least 10 animals.
  4. At least three separate replicates should be completed. Statistical analysis should be performed using the Student’s *t*-test.
*Note: Data should be presented as the average sperm number per animal (a mean value of 30+ individual counts across three or more separate replicates).*
- Size assay
  1. Score only non-activated, circular spermatids that are in focus.
  2. Each replicate should consist of at least 100 useable spermatids.
  3. Using ImageJ’s oval tool, hold shift to ensure circularity and outline and measure the area of spermatids that meet the inclusion criteria.
  4. At least three separate replicates should be completed. Statistical analysis should be performed using the Student’s *t*-test.
*Note: Data should be presented as the average spermatid size (a mean value of 300+ individual spermatids across three or more separate replicates).*
- Activation assay
  1. Using images of both focal planes for each field of view, score individual spermatids as activated (with pseudopod) or not activated (no pseudopod, including cells that are in the process of becoming activated with spiked or lobed projections).
  2. Each replicate should consist of at least 100 scored spermatids.
  3. For each condition, calculate the percentage of activated spermatids (out of total scored).
  4. At least three separate replicates should be completed.
  5. Calculate the average activation percentage per replicate and compare the samples using the Student’s *t*-test.
*Note: Data should be presented as the average activation percentage (a mean value of three or more percentages values, one per replicate).*
- Qualitative mitochondrial morphology assay
  This is a qualitative assay.
- Quantitative mitochondrial fusion assay
  1. Score z-stacks taken with the AlexaFluor546 filter. Use z-stacks to determine and score whether mitochondria are spherical (not fused) or oblong (fused).
  2. Each replicate should consist of at least 40 individual spermatids.
  3. For each condition, calculate the percentage of fused (out of total scored) mitochondria.
  4. At least three separate replicates should be completed.
  5. Compare data using the Student’s *t*-test.
*Notes:*
    - *Data should be presented as the average percentage of fused mitochondria per spermatid (a mean value of 90+ percentages across three or more separate replicates).*
    - *Changes in membrane potential can be determined using the fluorescence ratio of red to green channels using the JC-1 dye. ImageJ can be used for fluorescence analysis.*

## Recipes

1. Nematode Growth Medium (NGM) plates
  - Prepare stock solutions of 1 M MgSO_4_, 5 mg/ml cholesterol, 1 M KH_2_PO_4_, 1 M CaCl_2_, and 2.5% (w/v) streptomycin.
    - Dissolve 120.366 g of MgSO_4_ per 1 L water (1 M). Filter sterilize.
    - Dissolve 5 g of cholesterol per 1 L 100% ethanol (5 mg/ml). Store at 4 °C.
    - Dissolve 136.086 g of KH_2_PO_4_ per 1 L water (1 M). Filter sterilize.
    - Dissolve 110.98 g of CaCl_2_ per 1 L water (1 M). Filter sterilize.
    - Dissolve 25 g of streptomycin sulfate per 1 L water (2.5%). Filter sterilize. Store at 4 °C.
  - Add 3 g of NaCl, 17 g of agar, 2.5 g of peptone, and 950 ml of water to a glass flask. Include a stir bar.
  - Cover with foil and autoclave to sterilize and dissolve agar.
  - Cool, with stirring, to 55-60 °C.
  - Add 1 ml of 1 M MgSO_4_, 1 ml of 5 mg/ml cholesterol, 25 ml of 1 M KH_2_PO_4_, 1 ml of 1 M CaCl_2_, and 7.5 ml of 2.5% streptomycin.
  - Stir 5 min.
  - Dispense 11 ml into 6 cm Petri dishes using sterile technique.
  - Allow to solidify overnight.
  - Seed stock NGM plates with 250 μl of OP50-1 overnight culture (grown in LB + streptomycin, without shaking).
*Note: Mitotracker staining (Qualitative mitochondrial morphology assay) requires 25× concentrated bacteria. To make this, inoculate OP50-1 in 500 mL of LB + streptomycin. Grow with shaking, overnight at 37 °C. Centrifuge 20 min 5,000 × g at 4 °C (repeat as necessary to pellet bacteria from entire culture). Resuspend in sterile M9. Bring volume to 20 ml. Store at 4 °C for up to 6 months.*
  - Allow plates to dry (covered) 2-3 days before use.
2. Phosphate buffered saline (PBS)
  - Add 26.5 g of Na_2_HPO_4_·7H_2_O, 80 g of NaCl, 2 g of KCl, and 2 g of KH_2_PO_4_ to a glass bottle.
  - Bring volume to 1 L.
  - Sterilize by autoclaving.
3. PBS + 0.01% Triton X-100 (PBST)
  Using a cut pipette tip (to broaden opening), add 100 μl of Triton X-100 to 1 L sterile PBS. Mix well (do not shake).
4. SM Buffer (dextrose)
  - Prepare stock solutions of 1 M HEPES, 1 M NaCl, 1 M KCl, 1 M CaCl_2_, 1 M MgSO_4_, and 1 M dextrose.
    - Dissolve 238.3 g of HEPES per 1 L water (1 M). Filter sterilize.
      Although not necessary at this point, adjusting the pH to 7.8 before bringing solution to the final volume and sterilizing will expedite step 4c.
    - Dissolve 58.4 g of NaCl per 1 L water (1 M). Filter or autoclave to sterilize.
    - Dissolve 74.6 g of KCl per 1 L water (1 M). Filter or autoclave to sterilize.
    - Dissolve 110.98 g of CaCl_2_ per 1 L water (1 M). Filter or autoclave to sterilize.
    - Dissolve 120.366 g of MgSO_4_ per 1 L water (1 M). Filter or autoclave to sterilize.
    - Dissolve 180.2 g of dextrose per 1 L water (1 M). Filter or autoclave to sterilize.
  - Combine 2.5 ml of 1 M HEPES, 2.5 ml of 1 M NaCl, 1.25 ml of 1 M KCl, 250 μl of 1 M CaCl_2_, 50 μl of 1 M MgSO_4_, 500 μl of 1 M dextrose, and approximately 20 ml of water.
  - Adjust pH to 7.8.
  - Bring volume to 50 ml. Filter sterilize.
  - Store at 4 °C. Allow aliquots to come to RT before use.
5. SM Buffer (BSA)
  - Prepare stock solutions of 1 M HEPES, 1 M NaCl, 1 M KCl, 1 M CaCl_2_, 1 M MgSO_4_, and 5% (w/v) BSA.
    - Dissolve 238.3 g of HEPES per 1 L water (1 M). Filter sterilize.
      Although not necessary, adjusting the pH to 7.8 before bringing the solution to the final volume and sterilizing will expedite step 5c.
    - Dissolve 58.4 g of NaCl per 1 L water (1 M). Filter or autoclave to sterilize.
    - Dissolve 74.6 g of KCl per 1 L water (1 M). Filter or autoclave to sterilize.
    - Dissolve 110.98 g of CaCl_2_ per 1 L water (1 M). Filter or autoclave to sterilize.
    - Dissolve 120.366 g of MgSO_4_ per 1 L water (1 M). Filter or autoclave to sterilize.
    - Dissolve 2.5 g of BSA per 50 ml water (5% w/v). Filter to sterilize. Store at 4 °C.
  - Combine 2.5 ml of 1 M HEPES, 2.5 ml of 1 M NaCl, 1.25 ml of 1 M KCl, 250 μl of 1 M CaCl_2_, 50 μl of 1 M MgSO_4_, 1 ml of 5% (w/v) BSA, and approximately 20 ml of water.
  - Adjust pH to 7.8.
  - Bring volume to 50 ml. Filter sterilize.
  - Store at 4 °C. Allow aliquots to come to RT before use.
6. M9
  - Add 30 g of KH_2_PO_4_, 60 g of Na_2_HPO_4_, 50 g of NaCl, and 120 mg of MgSO_4_ to a glass bottle. Bring volume to 1 L.
  - Sterilize by autoclaving.
    Precipitation of calcium salts may occur after autoclaving. Re-dissolving may take several days. Agitation helps.
7. DAPI stock solution
  Dissolve 1 mg/ml in DMSO.
  - Protect from light.
  - Store aliquots at -20 °C. Can be thawed and re-frozen.
8. Pronase working solution.
  Dissolve 10 mg/ml of Pronase in water.
  Make fresh before each use.
9. MitoTracker stock solution.
  Dissolve 0.53152 g/ml of MitoTracker in DMSO (1 mM).
  - Protect from light.
  - Store in small aliquots at -20 °C. Minimize freeze-thaw cycles.
10. JC-1 stock solution
  Dissolve 1 mg/ml of JC-1 in DMSO (1.5 mM).
  - Protect from light.
  - Store in small aliquots at -20 °C. Minimize freeze-thaw cycles.
